# Control System for Automated Technological Process of Hot Stamping—A Case Study

**DOI:** 10.3390/ma16103658

**Published:** 2023-05-11

**Authors:** Ireneusz Wróbel, Piotr Danielczyk

**Affiliations:** Faculty of Mechanical Engineering and Computer Science, University of Bielsko-Biala, 43-309 Bielsko-Biala, Poland; pdanielczyk@ath.bielsko.pl

**Keywords:** hot forming, fault diagnosis, digital twin, Industry 4.0

## Abstract

Hot forming process has been used more and more frequently in the production of body structures of modern ultralight passenger cars for several years. This, unlike the commonly used cold stamping, is a complicated process, combining heat treatment and plastic-forming methods. For this reason, permanent control at each stage is required. This includes, among others, measurement of the blank thickness, monitoring its heating process in the suitable atmosphere in the furnace, control of the forming process itself, measurement of shape-dimensional accuracy as well as mechanical parameters of the finished drawpiece. This paper discusses the method of controlling the values of production parameters during the hot stamping process of a selected drawpiece. For this purpose, digital twins of the production line and the stamping process, made in accordance with the assumptions of Industry 4.0, have been used. Individual components of the production line with sensors for monitoring process parameters have been shown. The system’s response to emerging threats has also been described. The correctness of the adopted values is confirmed via tests of mechanical properties and the assessment of the shape-dimensional accuracy of a drawpiece test series.

## 1. Introduction

The challenges arising from the sustainable development policy [1], primarily concerning the reduction of CO_2_ emissions, are related to a large extent to the automotive industry. This in turn is associated with energy saving and the effective use of natural resources. Actions taken in this area should have a direct impact on reducing exhaust emissions of vehicles powered by traditional drive systems, as well as stimulate the rapid development of electric vehicles [2]. For this reason, efforts have been made to reduce the weight of passenger car bodies, while meeting all the required safety standards [3]. Therefore, the production of parts of load-bearing structures of modern car bodies forces the use of new construction materials and new technologies for their production.

**Figure 1:**
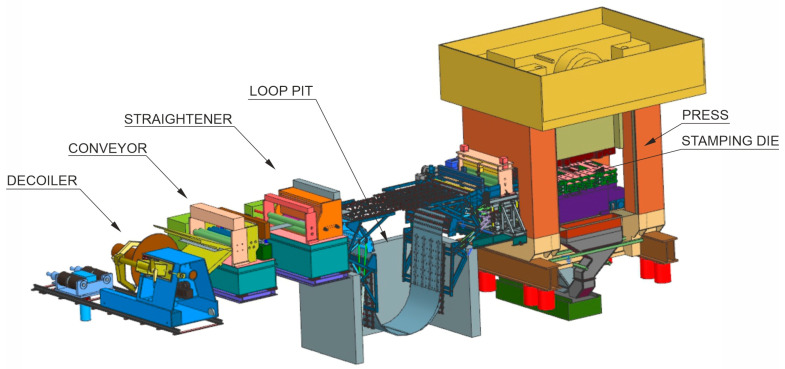
Scheme of cold forming process.

The dominant technology for manufacturing parts for the needs of the automotive industry is the stamping process [4,5]. In commonly used cold stamping processes, the production of ultralight components included in load-bearing structures of a car body is possible thanks to the use of DP (*Dual Phase* steels) with a tensile strength of up to 1000–1200 MPa [6,7]. Owing to such good strength parameters, while maintaining appropriate formability, it is possible to make sheet metal drawpieces of reduced thickness, without damaging the mechanical properties of a ready drawpiece. The cold pressing process takes place at a temperature close to the ambient temperature. The material in the form of sheet metal, wound on a coil is fed from the decoiler through the conveyer to the straightening device (Figure 1). Next, after passing through a series of straightening rollers, the material is collected in a loop pit. In the next stage, the sheet is transported to the press with a stamping die mounted on its table. After undergoing subsequent trimming and forming operations, the drawpiece is ready. Depending on the complexity of the product, the dies are made as progressive or transfer dies. In the first case, during the entire technological manufacturing process, a drawpiece is connected to the steel belt. In transfer dies, the product is moved between operations with the use of special grippers. It should be clearly emphasized that the quality of the drawpiece in the cold pressing process (shape and dimensional accuracy) is primarily influenced by the perfection of the die design. Strength properties of the finished product result from the properties of the material that is to be used for production. At this point, it is worth mentioning the unfavourable phenomenon of return springing of drawpieces which is particularly difficult to control during the production of DP steel components. This is one of the reasons for the increasing use of hot stamping technology.

**Figure 2:**
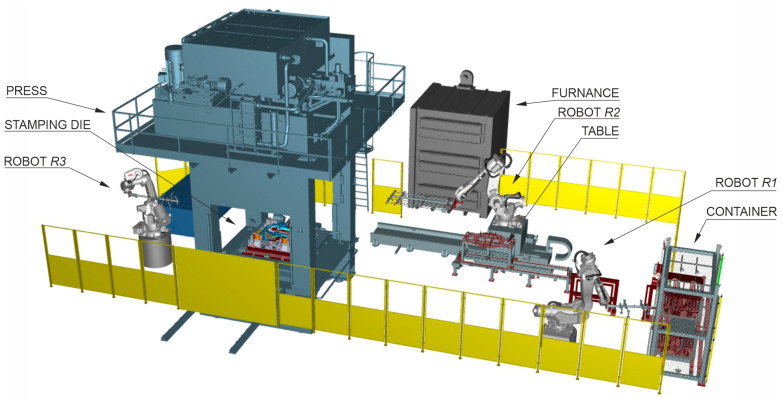
The scheme of hot forming line.

For over a dozen years, more and more body elements of new car models have been produced from manganese-boron steels [8,9,10,11,12] in the hot stamping technology [13]. After the hot stamping process, the material can achieve a tensile strength of up to 1900 MPa [14], which is a completely new quality in the production of high-strength drawpieces. In addition, this technology allows the production of drawpieces with areas of different thicknesses and, thus, with different mechanical properties in these areas [15,16]. In the process of cold pressing, obtaining such an effect is extremely difficult. During hot stamping, the drawpiece is shaped and quenched at the same time. The material changes from a ferritic–pearlitic structure to a martensitic structure. In order to achieve this result, a high cooling rate of at least 27 K/s is required. This forces the production of a tool with a special design with appropriately selected cooling channels [17,18,19]. The process itself is much more complicated than the cold pressing process, both in terms of the organization of the production line and the production technology of the drawpiece. For this reason, when preparing production with this technology, a digital twin [20] of the production line (Figure 2) is built in accordance with the assumptions of Industry 4.0 [21,22]. The twin is used to analyse the entire process of producing drawpieces.

During this process, properly prepared blanks are taken from a special container by the *R*1 robot’s arm and fed to the table, from which the *R*2 robot transfers them to the furnace. There, the blank is heated to the temperature of about 900–930 °C (austenitisation temperature), required for the proper drawpiece quenching. Next, the same robot transfers the heated blank from the furnace to the stamping die, and the forming stage begins. The tools are closed, and the pressing force of the press is significantly increased to the acceptable value in order to improve the conditions of heat exchange between the product and the working surfaces of the tools. The quenching process begins. In the next stage, the finished product is collected from the die by the *R*3 robot arm. Depending on the organization of the production line, the drawpiece is placed in a container or transferred directly to the control and measurement station by a belt conveyor. All activities must be properly synchronized, and production should take place in the shortest time possible. This is a big challenge for designers of such a process.

The quality of the drawpiece obtained in the hot stamping process should be understood not only as maintaining appropriate shape and dimensional accuracy but also as achieving appropriate mechanical properties of the product in the process of stamping and quenching. The final properties of the finished drawpiece are influenced by many more factors than in the case of cold stamping. These factors include the temperature and time of heating the blanks in the furnace, the time of transferring the blanks to the die, the temperature at the beginning of the stamping process, the quenching time, and the final temperature of the drawpiece.

The main task of the systems for monitoring and diagnosing stamping processes is to ensure the correct and stable course of the manufacturing process, which will allow the production of drawpieces of repeatable quality. Monitoring cold pressing is primarily about preventing critical situations that lead to die damage. Inductive sensors are installed in progressive dies. Their task is to control if the sheet metal strip is moved by the appropriate stroke value in each press cycle. In transfer dies, optical sensors are used to check whether the drawpiece is transferred by grippers to the next operation. If, in the transfer process, the drawpiece is made of a pre-punched sheet of metal, thickness control is additionally performed to prevent accidentally glued sheets of metal from being fed into the die. In addition, in special cases, laser sensors are used to check whether the waste generated in the cutting process is properly discharged. If additional operations are performed in the stamping process, e.g., threading operations, pressure sensors check if the tap is not broken. This also applies to punches that punch holes of small diameters.

**Figure 3:**
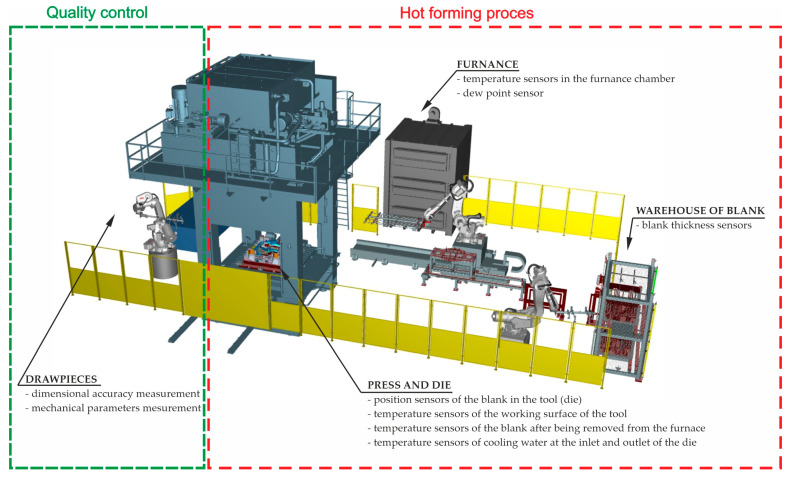
Monitoring systems of the hot forming process.

Monitoring key process parameters during hot stamping is a completely different issue. Here, as noted earlier, the quality of the final product is influenced by many more factors that must be constantly controlled. The first group of process control systems (Figure 3) consists of measurement systems cooperating with the automation of the production line. They control the process parameters in real time, and based on the measured values, an adequate response of individual machines operating on the line is taken. This group includes the blank thickness sensor, temperature sensors in the furnace chambers, dew point sensors of the furnace atmosphere, sensors of the blank position in the tool, temperature sensors of the working surfaces of the tool, temperature sensors of the blank after being removed from the furnace, and temperature sensors of cooling water at the inlet and outlet of the stamping die. The second group of measurement systems includes systems for controlling the quality of drawpieces. Optical measurement of the shape-dimensional accuracy of the produced drawpiece and measurement of the mechanical properties of drawpieces using non-destructive testing devices are performed here.

The vast majority of publications dealing with issues of stamping process control refer to monitoring selected parameters during the cold forming process. Some attention is given to the measurement of stamping forces. The results of these measurements are often treated as a reference point for the validation of numerical simulations, or they are used to control the uniformity of pressure loads in the die, as described in [23]. Monitoring pressure loads and the deep drawing process with the use of a system of sensors integrated with the tool is described in [24]. In [25], an attempt was made to monitor stamping processes using acceleration sensors with a bi-spectral analysis of the measurement results. Due to the ease of obtaining such a signal and the low cost of installing the sensor, this method was considered promising. The use of machine learning techniques to control the stamping process is presented in, among others, [26,27]. The method of detecting defects in drawpieces using vision systems is presented in, among others, [28].

Very few publications address this topic in relation to hot stamping processes. An extensive study [29] presents an overview of issues related to the construction and operation of modern stamping dies. These are of general nature and concern many aspects, e.g., materials used for the construction of tools, methods of working surface treatment, assessment of tool durability, and methods of their regeneration. In particular, part of the work was devoted to monitoring systems for stamping processes, including hot stamping, and their control based on the collected measurement data. The concept of a real-time monitoring system for hot stamping process parameters is presented in [30]. The system is designed to collect and process data from sensors installed on individual line elements. These data are stored in the database and are the basis for further analysis, thanks to which it is possible to forecast possible faults or failures. Article [31] presents a method of solving the problems related to the temperature control of the blank heated in the furnace to about 800–900 °C and the drawpiece at the temperature of 100–200 °C. The temperature was measured using a thermal imaging camera. A special algorithm was proposed that solves the problem of different settings of the thermal imaging camera for measuring temperatures with a large difference. Work [32] presents a system for monitoring acoustic emission in the hot stamping process. This system is designed to track the condition of the die and punch and detect the beginnings of their wear.

The main goal of the work is to prepare a control system for an experimental hot stamping line intended for the production of drawpieces in PHS (*Press Hardened Steels*) technology. As part of the conducted studies, the most important technological parameters affecting the process and the method of measuring these values with the use of appropriate sensors and measurement systems were selected. Acceptable limits of their variability and the method of system reaction (production line control algorithm) to any deviations from the set values were determined. Initial values of production parameters were chosen via performing a series of simulations of the stamping process using the Finite Element Method as well as the knowledge and experience of engineers and specialists in hot forming. The layout of the line, from the moment of taking the blank from the warehouse to the moment of transferring the finished drawpiece for measurement, was designed using digital twins of the process. The correctness of the line operation was assessed on the basis of tests of a trial series of drawpieces. These tests include testing mechanical properties such as hardness—HV (Vickers scale), yield strength—Rp0.2 and strength limit—Rm, as well as measurements of shape and dimensional accuracy. In addition, microstructure tests and long-term measurements of hydrogen embrittlement of the drawpieces will be performed in a four-point bending test. The novelty of this study is a comprehensive approach to the hot stamping process control. It includes the selection of initial values of technological parameters by simulation of the stamping process, determination of the method of measuring these parameters on the real production line, as well as detailed quality control of the produced drawpieces.

## 2. Measuring Systems Used for Controlling Hot Forming Process

This section will discuss the measurements that monitor the pressing process itself. In some cases, especially for those less common in industrial practice, the principle of their operation will be presented; in the case of dew point measurement, the motivation for this measurement will also be presented. In other cases, their role in the process will be indicated.

### 2.1. Measurement of the Blank Thickness

During the operation of the automatic hot stamping line, pre-prepared flat pieces are taken from the warehouse. The first sensor mounted on the line is the thickness sensor. It measures the thickness of the sheet, preventing two blanks glued together from entering the production process. Such a situation is unacceptable. Potential placement of more than one form in the die may, at best, damage the working surfaces of the dies and punches or lead to the destruction of the tool. Two types of sheet thickness sensors are used on hot stamping lines: ultrasonic sensors and optical sensors. The former, commonly used in many fields for several decades, operate based on measuring the speed of ultrasonic wave propagation in the tested material. The scope of their application is very wide, ranging from measuring the thickness of various materials, including multi-layer materials, through measuring the thickness of varnished coatings to detecting damage or discontinuities caused by, e.g., corrosion pitting in places inaccessible for other methods.

**Figure 4:**
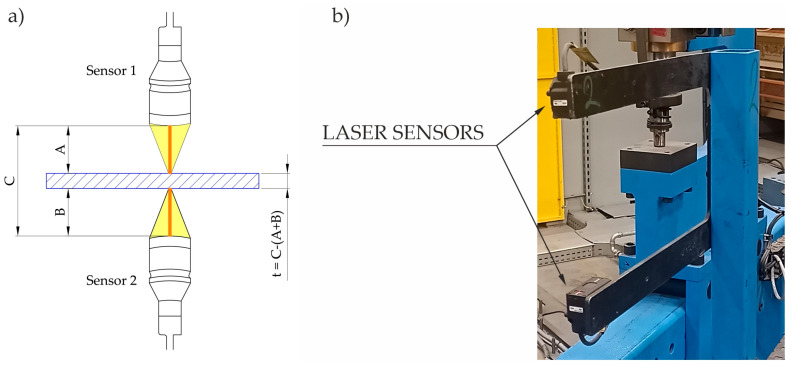
Measurement of blank thickness; (**a**) principle of sensor operation; (**b**) sensors mounted on the line.

Currently, modern optical sensors are used much more frequently. They perform measurements based on the principle of optical triangulation. A laser diode (class 2 laser) illuminates a point on the surface to be measured. The light reflected from this point is directed to a photosensitive matrix, where it is processed into a distance dimension. Most often, sheet thickness measurement is carried out using the differential measurement method (Figure 4). It consists of coupling two synchronously operating sensors and subtracting the values measured by these sensors from the fixed distance between them. Thanks to this solution, when the measured object moves within the measuring field, the measured value does not change.

The signal from this sensor affects the way the *R*1 robot works (Figure 2). If the sensor indicates too much thickness of the blank, the blank is put back into the container. Otherwise, it goes to the table operated by *R*2 robot and is loaded into the furnace.

### 2.2. Measurement of Temperature in the Furnace

Thermocouples installed in each of the three chambers of the furnace are used to control the temperature of the furnace in which the forms are heated to the austenitising temperature.

The values obtained from the thermocouples are used to control the operation of the heaters responsible for maintaining the required temperature in the chamber. This is usually a two-stage control with fixed temperature thresholds. When the measured temperature in the chamber drops below 890 °C, the heaters are switched on and heat the furnace chamber. When the temperature exceeds 930 °C, the power of the heaters is turned off.

**Figure 5:**
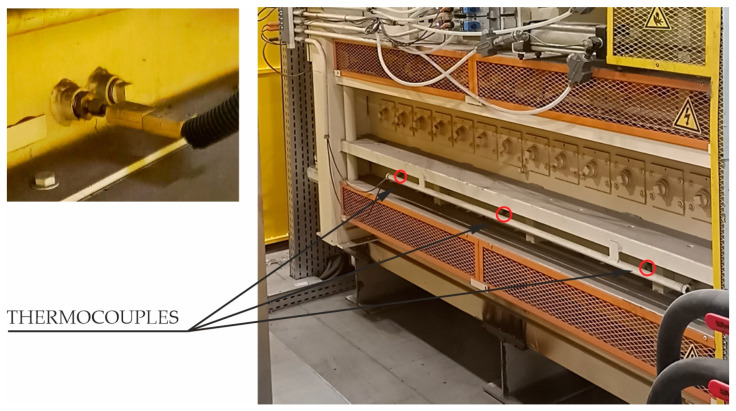
Measurement of temperature in chamber of the furnace.

Figure 5 shows the location of the thermocouples installed in one of the furnace chambers in the discussed hot stamping line.

If it is not possible to maintain the temperatures in the furnace within the required range of 890–930 °C, the process is stopped. This indicates a failure of the furnace.

### 2.3. Measurement of Dew Point Temperature of Furnace Atmosphere

When the blanks are heated, an undesirable phenomenon of hydrogen diffusion to the structure of steel used in hot stamping processes (22MnB5) may occur. This may lead to the formation of the so-called hydrogen embrittlement of drawpieces [33,34,35]. As a result of increasing the hydrogen content in the finished drawpieces, local stresses may arise, causing the loss of structural cohesion. The effects of hydrogen are long-lasting. Undesirable cracks in the drawpieces may occur shortly after production, during the assembly of the bodywork, as well as during the operation of the car. Manufacturers of modern vehicles built from elements produced in the PHS technology recognise this problem and expect that the causes of hydrogen embrittlement will be eliminated already at the production stage. This is related to limiting the amount of hydrogen diffusing into the material during the heating of the blanks.

After opening the furnace door in order to take a heated blank or load a blank for heating, the air from the furnace mixes with the ambient air. To reduce the amount of water vapour in the furnace atmosphere, dried air is blown into the chamber. It is estimated that in order to significantly reduce the risk of hydrogen embrittlement of the manufactured products, it is necessary to maintain an air atmosphere in the furnace with a degree of humidity of about 0.0025 kg H_2_O/kg of dry air. This corresponds to the partial pressure of water vapour of about 400 Pa (dew point equal to −5 °C). Such a requirement is included in the standards of car manufacturers for the quality of drawpieces.

**Figure 6:**
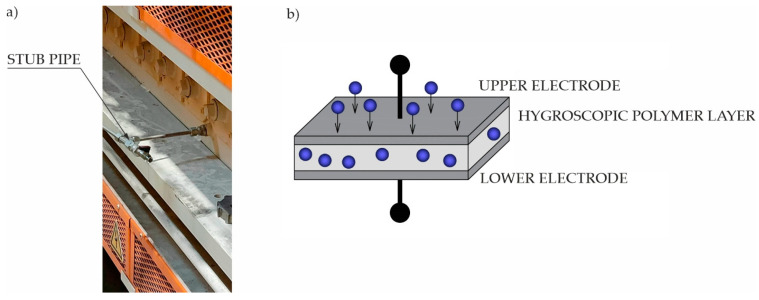
Measurement of dew point temperature; (**a**) air sample for measurement; (**b**) operational scheme of sensor.

The dew point temperature is measured for the air taken from the furnace using a special stub pipe (Figure 6a), from where it goes to the capacitive humidity sensor (Figure 6b).

Such a sensor is equipped with two electrodes separated from each other by a thin layer of hygroscopic polymer acting as a dielectric. The change in the dielectric constant of the hygroscopic material is proportional to the measured relative humidity of the air. By additionally measuring the air temperature, the dew point temperature is determined. If its value is greater than the admissible value, the flow of dried air to the furnace chamber is increased.

### 2.4. Measurement of the Blank Temperature

**Figure 7:**
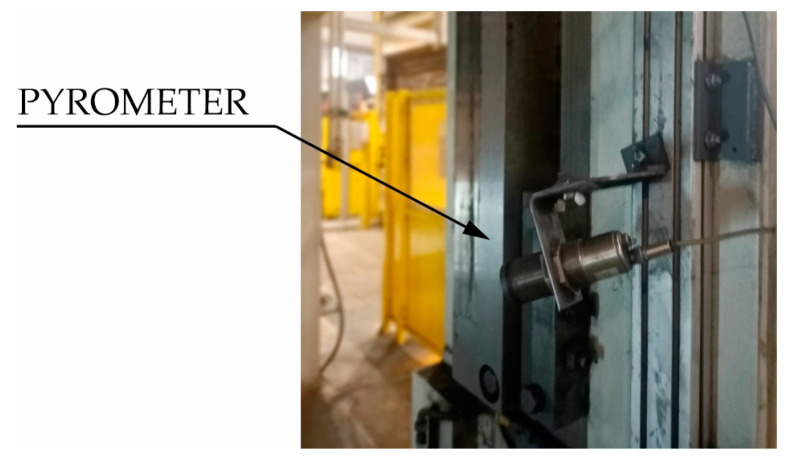
Pyrometer for measuring temperature of the blank.

The first measurement performed before the stamping process starts is the measurement of the temperature of the blank. For this purpose, a pyrometer mounted on the body of the press is used (Figure 7). When the measured temperature of the blank is lower than 780 °C, which may result in incorrect quenching process, the automation controlling the hot stamping line sends information to *R*2 robot (Figure 2) responsible for transferring the heated blank from the furnace to the die to put the blank to a scrap container. Drawpiece buyers, such as large car concerns, do not allow the possibility of reusing such material. Al-Si coatings, which cover blanks after reheating, grow excessively. This causes, among other things, problems when spot-welding of drawpieces. The details are discussed in [36,37].

### 2.5. Measurement of Positioning of the Blank on the Stamping Die

**Figure 8:**
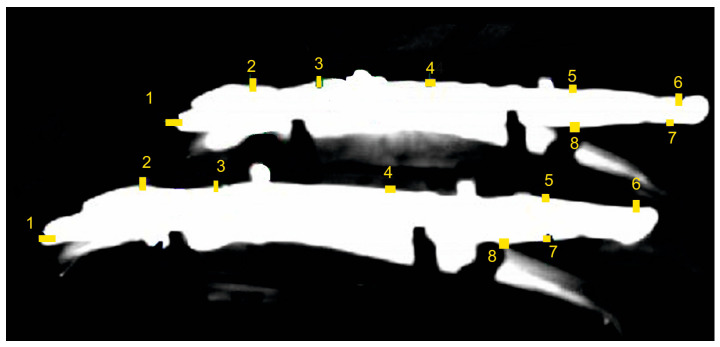
Measurement of the blank position in the tool (1–8 position measurement points).

After placing the blank on the die, the thermal imaging camera verifies the accuracy of its positioning. The robot transfers the blank from the furnace to the die and puts it down when the base hole of the blank is above the tool’s base pin. This ensures proper placement of the blank before the forming process begins. There is a risk of placing the blank on the tool incorrectly, for example by improperly picking up the blank from the furnace. This situation can lead to permanent and costly damage to the tool components. In order to prevent such situations, control systems, which analyse the image from the thermal imaging camera, are used. When the camera receives a signal from the control system about placing the form on the die, it takes a picture (in this case, a monochrome one). The system, based on the previously defined eight measuring points (Figure 8), measures the shortest distance between these points and the detected edges of the blank. When the calculated distance values are within the set tolerances, the line control system allows the press punch to move down, and the stamping process begins. Otherwise, the line is stopped, and a piece position error signal appears. The blank is manually removed from the die, and the production process is restarted.

### 2.6. Temperature Measurements of the Tools after the Stamping Process

**Figure 9:**
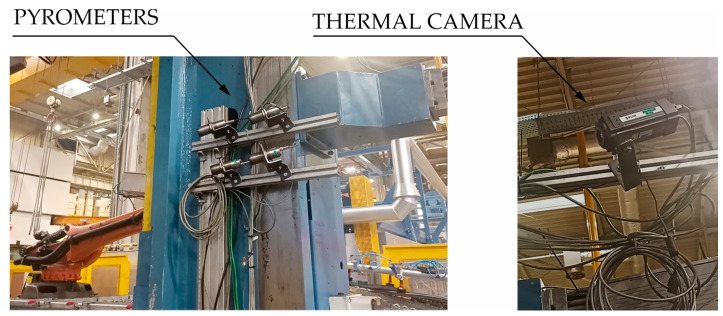
Measurement of temperature in the stamping die.

In the stamping process, it is very important to control the temperature of the die and punch after removing the drawpiece from the tool. A thermovision camera and a set of pyrometers are used to measure the temperature of the tools after the stamping process (Figure 9).

After removing the finished drawpiece from the die, the system controlling the automated production line sends the signal to thermal imaging camera, which measures temperature of working surfaces of the die and the punch. After the pressing process, these temperature values should not exceed 200 °C. In addition, in some cases, pyrometers are used to measure temperature in these places of the die where overheating points (so-called hot spots) were identified at the development stage of the hot stamping process and at the stage of its computer simulation. There the temperature can be up to 250 °C. Failure to meet these conditions may lead to production of drawpieces with incorrect parameters and adversely affect the operational life of the die, increasing its thermal wear.

**Figure 10:**
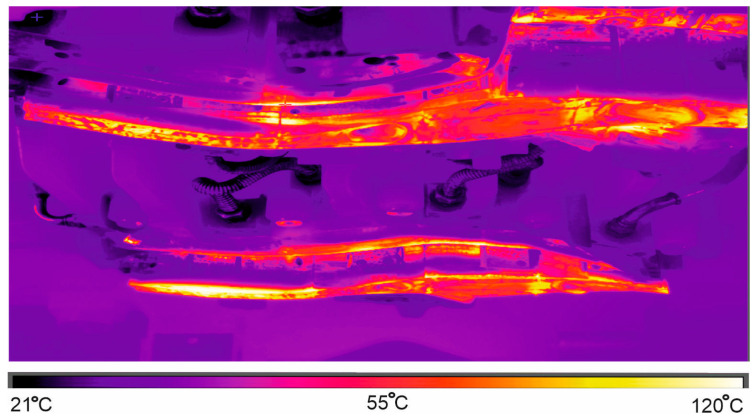
Image from thermovision camera.

Figure 10 presents an image from thermovision camera, displaying temperature of the punch after opening the die and removing the drawpiece.

After the measurement, the obtained image is analysed, and the area with the highest temperature is searched. When the value of this temperature does not exceed the limit, the production process is unchanged. Otherwise, the automatic control stops the production line until the maximum temperature on the working surfaces of dies and punches drops to acceptable values. It is also possible to lower these temperatures by increasing the flow of the cooling liquid through the die, as well as lowering its temperature in the cooling tower.

## 3. Measuring Systems Used to Control the Quality of Drawpieces

Systems for quality control of produced drawpieces can be divided into optical systems to control the shape and dimensional accuracy and non-destructive systems to control mechanical parameters such as yield strength Rp02, tensile strength Rm and hardness measured in HV degrees.

### 3.1. Control of Shape-Dimensional Accuracy of Drawpieces

Optical 3D scanners are used to control the shape and dimensional accuracy of the production line. Most often, these are robotic, specialised measuring systems, where the optical measuring head is mounted on the wrist of an industrial robot. These systems are resistant to vibrations, temperature spikes, do not require air-conditioned rooms, and are installed near production lines.

**Figure 11:**
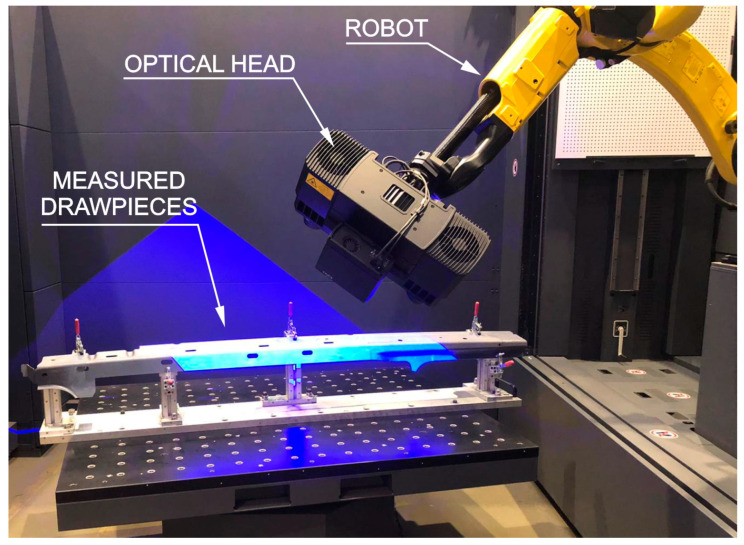
Automated measuring cell.

One of the solutions available on the market is the Zeiss automated measuring cell, as shown in Figure 11.

The robot performs a special measurement program, consisting in measuring the tested drawpiece from each side. The result of the measurement is a cloud of points describing the shape of the measured drawpiece. It is matched in RPS points (Reference Point System) to the CAD model of the drawpiece. Afterwards, deviations in the dimensions of the real object from the CAD model are calculated.

### 3.2. Control of Mechanical Properties of Drawpieces

**Figure 12:**
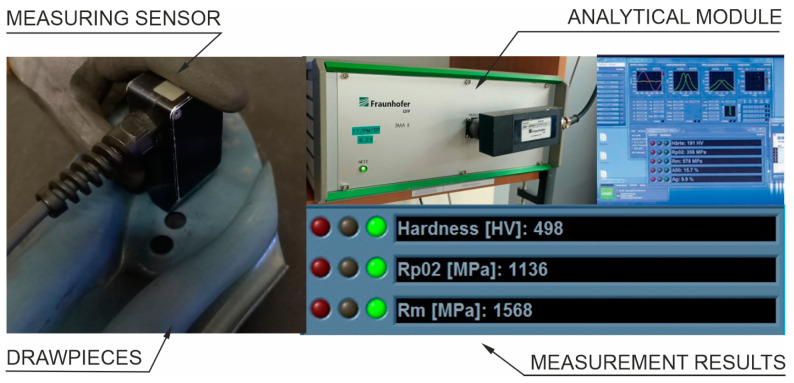
Measurement of mechanical parameters of the drawpiece.

Non-destructive tests are performed to control the mechanical parameters of the drawpieces (Rp02, Rm and HV hardness). The device used for this purpose is the 3MA device (Micromagnetic Multiparameter Microstructure and Stress Analysis) produced by the Fraunhofer Institute [38,39]. It consists of a measuring head and a module connected to a computer for analysing the signal coming from the head. This system analyses the magnetic properties of 22MnB5 steel and on this basis determines the mechanical parameters. Before the measurement, the sensor is calibrated to measure the parameters of a specific steel grade. The measurement consists of touching the surface of the measured drawpiece with the measuring head. Figure 12 shows the 3MA system measuring a drawpiece produced on the production line.

If drawpieces do not meet the set parameters, a number of control activities are undertaken. It begins with an inspection of the working surfaces of the dies and punches for possible damage. The surfaces are also checked for any residues of Al-Si coatings which are usually used to cover hot stamping sheets. Subsequently, the cooling system of the tool and the properties of the material for drawpieces are inspected. Additionally, the temperature parameters of the process undergo control. If necessary, the working surfaces of the tools are corrected.

## 4. Tests of the Hot Stamping Process Control System on an Experimental, Automated Production Line

**Figure 13:**
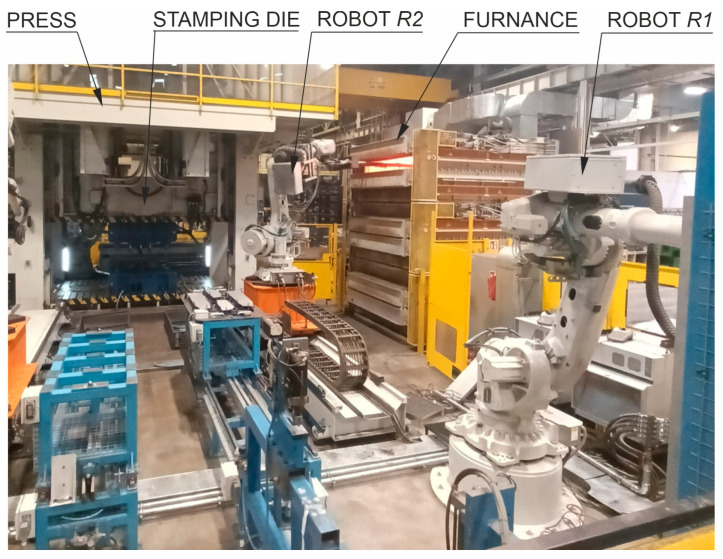
Experimental hot stamping line.

Figure 13 shows an experimental hot stamping line, whose model in the form of a digital twin (Figure 2) is discussed in point 1. The line is used to test new technical solutions developed as part of the study, to start the production of new drawpieces and to select optimal parameter values that can affect the stamping process. It is fully functional and can also be used as a production line.

**Figure 14:**
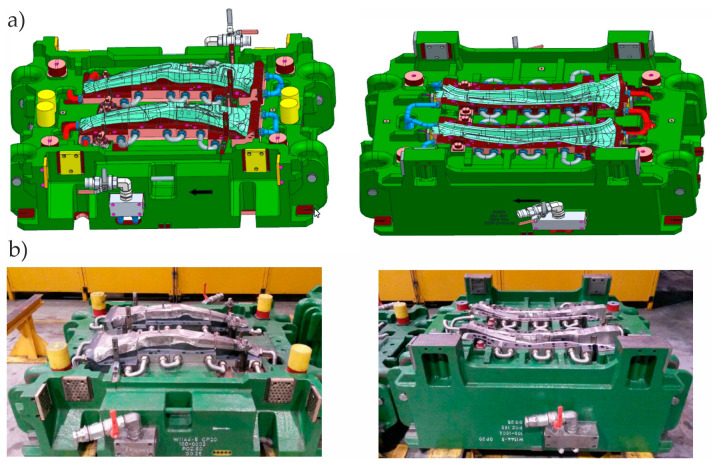
(**a**) CAD model of the tool; (**b**) tool selected for tests of the stamping process control system.

**Figure 15:**
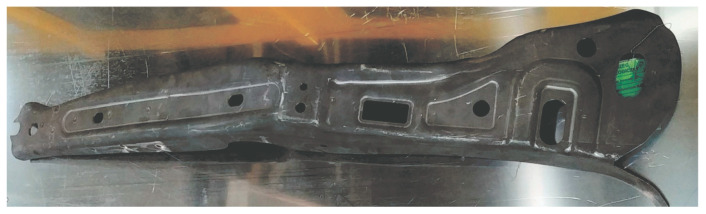
Drawpiece produced as part of the tests.

The hot stamping process control system was tested using a die (Figure 14) intended for the production of the B-pillar interior. The 2.2 mm thick drawpiece (Figure 15) was made of 22MnB5 steel. It is steel intended for hot stamping, during which the drawpiece is shaped and quenched at the same time. It is assumed that after the pressing process, the drawpiece will have a martensitic structure and the following mechanical parameters: yield point Rp0.2 = 950–1200 MPa, strength limit Rm = 1300–1650 MPa, and hardness HV = 400–550.

**Figure 16:**
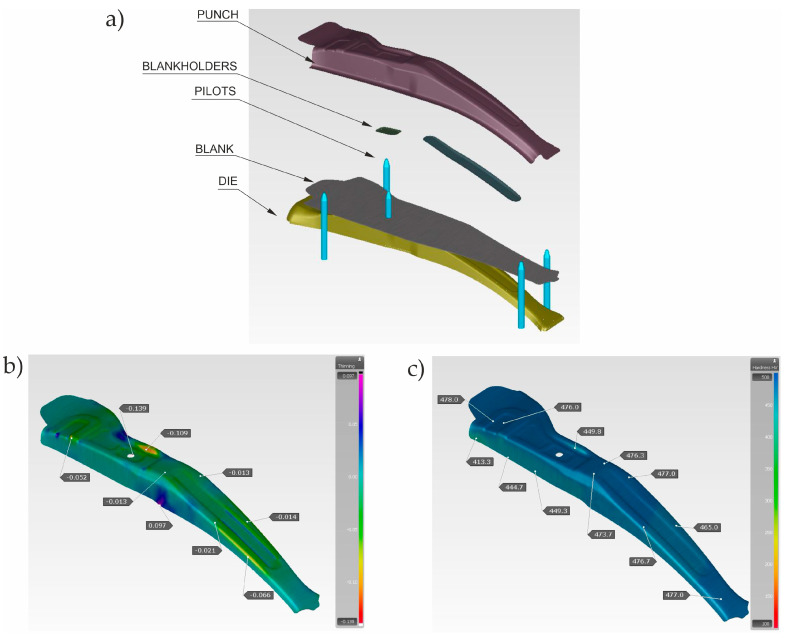
Results of hot stamping simulation; (**a**) 3D model of tools in the Autoform package, (**b**) amount of thinning, and (**c**) hardness.

During production trials, the following parameters of the technological process were controlled: the thickness of the blank, the temperature of the furnace chamber, the temperature of the atmosphere dew point in the furnace, the temperature of the blank before pressing, the location of the blank in the tool, and the temperature of the working surfaces. The initial values of these parameters and the permissible ranges of their variability (Table 1) were selected on the basis of the analysis of computer simulation results of the stamping processes carried out in the Autoform package supported by the experience of engineers. During the simulations, the impact of, among others, the temperature of the blank after taking it out of the furnace and the temperature of the tool on the feasibility of the product was tested. Most of all, the value of thinning in the drawpiece and its hardness were assessed. An example of the analysis result for the most favourable set of process parameters is shown in Figure 16.

All parameters recorded in Table 1 were measured via appropriate measuring systems, described in Point 2. The parameter values were analysed in real time via the PLC, which was programmed to react to exceeding their permissible values.

Tests in production conditions consisted of the production of four series of 200 drawpieces each and testing the correct operation of the hot stamping process control system. When starting the first series of production, the optical system for measuring the position of the blank in the die detected an incorrect positioning and stopped the entire production process. It was necessary to correct the working trajectories of the *R*2 robot responsible for transferring the blank from the furnace to the die. In addition, during the production of all four production series, the limit values for any of the defined process parameters were not exceeded.

## 5. Control of Mechanical Parameters and Shape and Dimensional Accuracy of Produced Drawpieces

From each of the four production series, five drawpieces were randomly selected and inspected. Mechanical parameters were measured non-destructively using a 3MA device, and the shape and dimensional accuracy were measured in an automated measuring cell using a GOM 3D scanner. In addition, steel microstructure was also checked on one randomly selected drawpiece from each production series.

### 5.1. Measurement of Mechanical Parameters Rp0.2, Rm, Hardness

**Figure 17:**
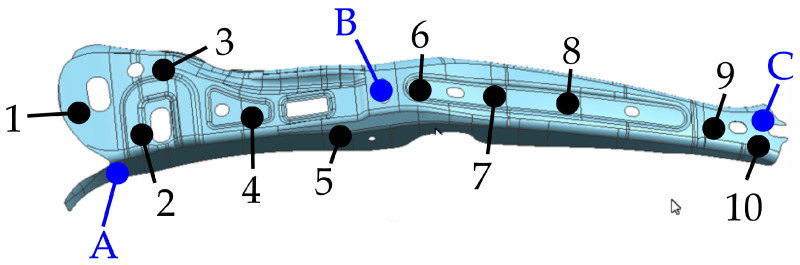
Measurement points (1–10 mechanical parameters measurement points, A,B,C—samples location).

Figure 17 shows the distribution of measurement points (from 1 to 10) where the yield strength and tensile strength, hardness were measured, and the distribution of areas (A, B and C) from which samples were taken for microstructure testing.

Table 2 presents the results of these measurements in the form of the maximum, minimum, average, and standard deviation of the measured values.

Analysing the data presented in Table 2, it can be concluded that at each measuring point of the interior of the B-pillar, the required mechanical parameters of the drawpiece were achieved. The hardness was within the assumed range of 400 to 550 HV. The yield point at each point ranged from 950 to 1200 MPa, and the strength limit reached 1300–1650 MPa.

### 5.2. Microstructure Analysis

**Figure 18:**
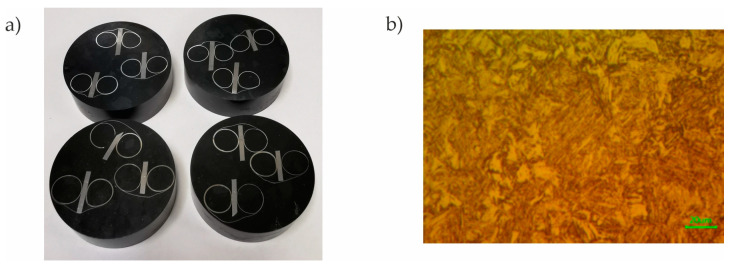
Measurement of the microstructure of the drawpiece material: (**a**) samples prepared for testing (**b**) microstructure.

From four randomly selected drawpieces (one from each production series), samples for microstructure analysis were cut using a water-abrasive cutter. The material prepared this way was positioned with a metallographic clip and then embedded in phenolic resin (Figure 18a). In the next stage, the samples were ground using diamond discs and polished using diamond slurries with a grain size of 9 µm and 3 µm and oxide slurries with a grain size of 0.05 µm. The material was etched in a 4% Nital-4 acid solution. The structure was checked at 50 times magnification using a microscope type: AmScope ME1200TC-5M Inverted Trinocular Metallurgical Microscope + 5MP Camera. Figure 18b shows the microstructure of the sample taken from the C measuring point (see Figure 17) of the drawpiece from the third production series.

The resulting structure is a martensitic structure required after the hot stamping process. It is characterized by high mechanical properties. Such a structure was observed in all samples taken from each of the measurement areas (A, B, and C) of each of the four drawpieces.

### 5.3. Measurement of Shape and Dimensional Accuracy

**Figure 19:**
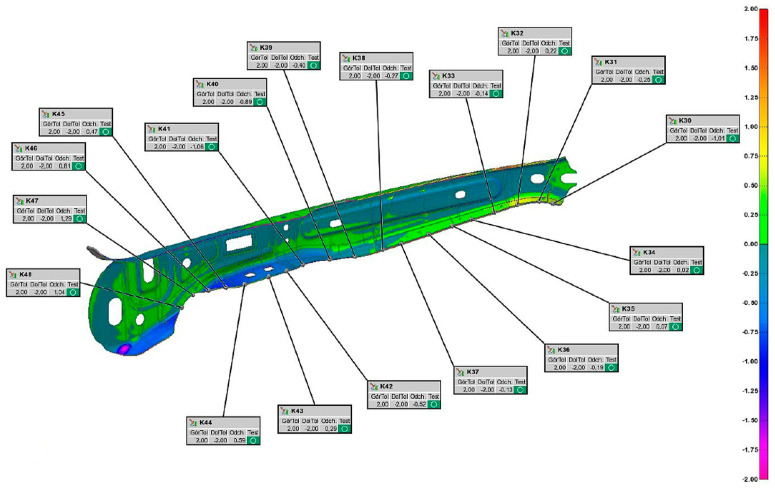
Drawpiece measurement report.

When starting to test the shape and dimensional accuracy of the produced drawpieces, the requirements defined in the technical documentation of drawpieces were first analysed. Based on this analysis, a measurement plan was prepared. From each of the four series of produced drawpieces, five were randomly selected and measured in an automated measuring cell. Figure 19 shows an example of a measurement report.

Analysing the measurement reports, it was found that none of the measurement points exceeded the assumed tolerances for the production of the drawpiece.

### 5.4. Measurement of Hydrogen Embrittlement in the Four-Point Bending Test

**Figure 20:**
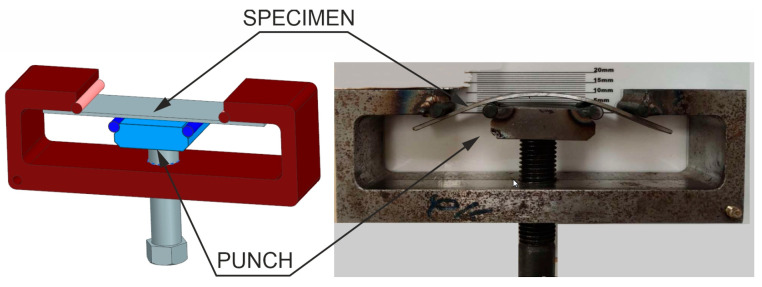
Measurement of hydrogen embrittlement.

As noted earlier, the phenomenon of hydrogen embrittlement can lead to uncontrolled cracking of car body drawpieces during the normal operation of cars. A simple workshop test of the material’s resistance to hydrogen embrittlement is four-point bending of samples taken from the drawpiece [40]. Such a rectangular sample with dimensions of 110 × 25 mm is placed in a special device (Figure 20).

The punch is tightened with a screw in such a way that the deflection of the sample causes stress equal to 80% of the yield strength. The sample is left in the instrument for 300 h and then inspected. No cracks or surface scratches, which could lead to cracking, were found in the tested sample. This means that the furnace atmosphere control system is working properly.

## 6. Conclusions

The complex measurement system for monitoring the hot stamping process and quality control of finished drawpieces presented for the test line is successfully used in one of the European stamping plants producing for the needs of the automotive industry. Continuous supervision of the process and control of drawpieces quality enables the production of repeatable parts in accordance with the requirements of large automotive concerns. As part of this study, a trial series of drawpieces was made to confirm the assumptions regarding the value of production parameters. Their correctness was confirmed by obtaining the mechanical parameters (Rp0.2, Rm, HV) consistent with the assumptions, the martensitic structure and the shape and dimension accuracy of the produced drawpieces. This means that the values of production parameters are selected correctly and do not cause undesirable changes in the shape of the drawpiece. Otherwise, uncontrolled hardening deformations (springback) occur, resulting from internal stresses generated during the heating and cooling of the drawpiece [41].

Implementation of the Industry 4.0 concept into industrial practice forces the expansion of this system. The aim of subsequent actions, in addition to process automation which is the pillar of Industry 4.0, should be the reduction in the number of drawpieces with incorrect parameters. For this reason, in the next stage of work, the presented control system for key process parameters will be equipped with tools for collecting and multi-criteria analysis of data from process sensors. This will allow the identification of the causes of drawpiece defects based on the analysis of past data, as well as those collected during production. An appropriately early reaction of the system to sensor readings that differ from the values adopted as a reference will enable counteracting emergency situations.

## Figures and Tables

**Table 1:** Initial values of process parameters.

No.	Name of Parameter	Nominal Value	Tolerance	Alarm Value
1	Blank thickness	2.2 mm	±0.1 mm	>2.3 mm
2	Furnace chamber temperature	930 °C	±10 °C	<880 °C
3	Dew point temperature	−6 °C	±1 °C	>−5 °C
4	Blank temperature	830 °C	±10 °C	<715 °C
5	Blank position	0 mm	±0.4 mm	>0.4 mm <−0.4 mm
6	Tool temperature	150 °C	±10 °C	>200 °C

**Table 2:** Measurement results of mechanical parameters for all measured drawpieces (MAX—maximum value, MIN—minimum value, MEAN—average value, SDV—standard deviation).

Point	1	2	3	4	5	6	7	8	9	10
Hardness HV, -										
MAX	518	513	533	515	533	510	502	506	513	518
MIN	498	485	481	481	451	450	458	463	458	464
MEAN	509	496	501	498	491	481	480	485	478	503
SDV	9	10	21	14	30	24	16	19	22	22
Yield strength Rp0.2, MPa										
MAX	1123	1094	1142	1108	1111	1111	1078	1090	1068	1133
MIN	1097	1066	1070	1075	953	965	1005	969	954	1018
MEAN	1115	1083	1099	1095	1066	1053	1056	1056	1037	1107
SDV	10	11	28	14	66	61	30	49	47	50
Tensile strength Rm, MPa										
MAX	1573	1529	1616	1540	1616	1545	1496	1528	1580	1580
MIN	1511	1487	1455	1478	1296	1310	1425	1329	1312	1388
MEAN	1552	1504	1523	1507	1491	1462	1474	1457	1447	1534
SDV	24	18	58	30	119	97	29	79	96	82

## Data Availability

Not applicable.
